# Evaluation of Serum Fibrinogen, Plasminogen, **α**2-Anti-Plasmin, and Plasminogen Activator Inhibitor Levels (PAI) and Their Correlation with Presence of Retinopathy in Patients with Type 1 DM

**DOI:** 10.1155/2014/317292

**Published:** 2014-04-10

**Authors:** Sefika Burcak Polat, Nagihan Ugurlu, Fatma Yulek, Huseyin Simavli, Reyhan Ersoy, Bekir Cakir, Ozcan Erel

**Affiliations:** ^1^Endocrinology and Metabolism Department, Ataturk Training and Research Hospital, Yildirim Beyazit University, 6800 Ankara, Turkey; ^2^Ophthalmology Department, Ataturk Training and Research Hospital, Yildirim Beyazit University, Ankara, Turkey; ^3^Ophtalmology Department, Izzet Baysal Government Hospital, Bolu, Turkey; ^4^Department of Biochemistry, Ataturk Training and Research Hospital, Yildirim Beyazit University, Ankara, Turkey

## Abstract

*Background.* Diabetic retinopathy (DR) is the leading cause of blindness in the world. Retinopathy can still progress despite optimal metabolic control. The aim of the study was to determine whether different degrees of DR (proliferative or nonproliferative) were associated with abnormally modulated hemostatic parameters in patients with T1DM. * Method.* 52 T1DM patients and 40 healthy controls were enrolled in the study. Patients were subdivided into three categories. Group I was defined as those without retinopathy, group II with NPRP, and group III with PRP. We compared these subgroups with each other and the control group (Group IV) according to the serum fibrinogen, plasminogen, alpha2-anti-plasmin (**α**2-anti-plasmin), and PAI. *Results*. We detected that PAI-1, serum fibrinogen, and plasminogen levels were similar between the diabetic and control groups (*P* = 0.209, *P* = 0.224, and *P* = 0.244, resp.), whereas **α**2-anti-plasmin was higher in Groups I, II, and III compared to the control group (*P* < 0.01, *P* < 0.05, and *P* < 0.001, resp.). There was a positive correlation between serum **α**2-anti-plasmin and HbA1c levels (*r* = 0,268, *P* = 0.031). *Conclusion*. To our knowledge there is scarce data in the literature about **α**2-anti-plasmin levels in type 1 diabetes. A positive correlation between **α**2-anti-plasmin with HbA1c suggests that fibrinolytic markers may improve with disease regulation and better glycemic control.

## 1. Introduction

Diabetes mellitus (DM) is a condition associated with propensity for thrombosis and increased risk of cardiovascular complications, leading to morbidity and mortality of diabetic patients [1]. Microvascular and large vessel diseases are distinct in regard to pathophysiology, and there is no current evidence to suggest that these two conditions share a common pathogenetic pathway. Diabetic retinopathy (DR) is the leading cause of loss of vision in the world and of new cases of blindness in patients aged between 20 and 64 years [1].

Although strict glycemic control and the regulation of blood pressure reduce microvascular complications, retinopathy and nephropathy can still progress in some patients despite optimal metabolic control. This suggests that factors other than hyperglycemia, such as abnormal hemostatic parameters, may play a role in the disease pathogenesis. Based on this hypothesis, several markers of hypercoagulation including fibrinogen, plasma activator inhibitor (PAI), and alpha-2-anti-plasmin have been identified. The hypercoagulable state can be explained by imbalance between levels of plasma procoagulant proteins and anticoagulant activity [2].

A positive association between increased levels of fibrinogen and the occurrence of microangiopathy was suggested. [3]. Myrup et al. demonstrated that fibrinogen levels were significantly higher in patients with nephropathy compared to those without [4]. Additional studies revealed that plasminogen and plasma activator inhibitor (PAI) levels were not affected in type 1 DM [5, 6], whereas coagulant activity was higher in patients with documented microvascular complications [7, 8]. Alpha 2-anti-plasmin (*α*2-anti-plasmin, or plasmin inhibitor) belongs to serine protease inhibitor (Serpin) family and it is responsible for inactivation of plasmin that is an important enzyme responsible for fibrinolysis and the degradation of various other proteins [9]. It was recently shown that there is a correlation between plasma plasmin-*α*2-anti-plasmin complex (PAP) and diabetic retinopathy regardless of other traditional risk factors for diabetic retinopathy, such as duration of diabetes, SBP, A1C, and serum creatinine levels [10].

The aim of the present study was to determine whether different degrees of DR (proliferative or nonproliferative) were associated with abnormally altered hemostasis in patients with type I DM. In addition, we planned to define whether there was a significant correlation between thrombotic tendency (as assessed by the levels of fibrinogen, PAI, plasminogen, and *α*2-anti-plasmin) and metabolic control.

Those parameters were compared in patients without retinopathy or with different degrees of retinopathy and the control group. To our knowledge, this is the first study that includes all these parameters and compares them between three different subgroups of type 1 diabetes patients containing relatively large numbers of patients.

## 2. Methods

### 2.1. Study Design

Fifty-two patients with type 1 diabetes (30 female and 22 male, with an age range of 17–63) who regularly visited our Endocrinology Clinics between January 2010 and December 2011 were enrolled in the present study. Each patient accepted and signed informed consent forms. Our local ethics committee approved the study. Medical history, current cigarette smoking, current alcohol consumption and drug history were recorded. Doppler analysis of vessels was used to detect the presence of macroangiopathy and venous or arterial thrombosis. Hypertension was defined as systolic blood pressure (SBP) > 140 mmHg, diastolic blood pressure > 90 mmHg, or the current use of antihypertensive medications. Resting blood pressure was measured three times in the seated position.

Patient selection was made by the same endocrinologist on the basis of the following inclusion criteria: type 1 DM (as defined by deficient c peptide secretion and autoantibody positivity at the time of diagnosis), duration of disease being longer than 5 years, normal blood pressure, normal serum triglyceride and LDL levels, and the absence of chronic kidney disease or peripheral obliterating arteriopathies, and other macrovascular disease. Forty healthy, nondiabetic subjects (20 male and 20 female), aged between 26 and 51 years, were recruited from hospital staff to serve as the control group.

Nephropathy was diagnosed by the presence of microalbuminuria (30–300 mg/day, Roche Diagnostics) or macroalbuminuria (>300 mg/day, Roche Diagnostics) in at least two samples of early morning urine collected over a 6-month period in the absence of infection, renal disease, or heart failure. Blood pressure and serum creatinine levels were measured to evaluate renal function.

All patients were treated only with subcutaneous insulin (human or analog). All test subjects were nonsmokers who had not taken drugs that could affect hemostasis for at least 4 weeks prior to the study.

### 2.2. Ocular Evaluation

All subjects underwent detailed ophthalmologic examination, including best-corrected visual acuity, applanation tonometry, anterior segment slit lamp biomicroscopy, and dilated fundus examination. Colored fundus photographs were taken from all subjects by nonmydriatic retinal camera (CR2-45 NM Nonmydriatic Fundus Camera, Canon Inc, Japan), and patients were then subdivided into three categories. Group I was defined as those without retinopathy, group II with nonproliferative retinopathy, and group III with proliferative retinopathy. Nonproliferative retinopathy (NPDR) was defined as nerve-fiber layer infarcts (cotton wool spots), intraretinal hemorrhages, hard exudates, and microvascular abnormalities (including microaneurysms, occluded vessels, and dilated or tortuous vessels) primarily in the macula and posterior retina. Proliferative diabetic retinopathy (PDR) was determined by the presence of neovascularization arising from the disc and/or retinal vessels and the resulting preretinal and vitreous hemorrhage, subsequent fibrosis, and traction retinal detachment.

### 2.3. Biochemical Evaluation

Blood samples were drawn into plain vacutainers from the antecubital veins of healthy controls and patients after 12 hours of fasting and 30 min of supine rest between 8 and 9 a.m. Glycated hemoglobin (HbA1c) was measured by high-performance liquid chromatography (HPLC), and the nondiabetic range used was 4.0–6.0%. To measure serum glucose and lipids (total cholesterol, triglycerides, and high density lipoprotein cholesterol), blood was drawn into evacuated siliconised tubes, and measurements were made by an autoanalyzer using enzymatic methods. The levels of plasma plasminogen, *α*2-anti-plasmin (Cusabio Biotech Co., Ltd), fibrinogen (MTI Tokra), and PAI-1 (Bender Med Systems, Vienna, Austria) were also measured.

### 2.4. Statistical Analysis

The SPSS version 11.5 software, Windows Edition (SPSS, Chicago, IL), was used for all statistical analysis. The nonparametric Kolmogorov-Smirnov test was used to compare samples with the reference probability distribution, whereas the homogeneity of the variances was assessed using Levene's test. Descriptive statistics are presented as means ± standard deviation (SD) and medians (minimum-maximum) for continuous variables and as percentages (%) for categorical variables. The significance of the differences between mean values was assessed by one-way ANOVA, while the Kruskal-Wallis test was used for median values. If significant differences were detected after one-way ANOVA or Kruskal-Wallis tests, post hoc Tukey's HSD or Conover's nonparametric multivariance comparison tests were used to define the effective factors. For categorical variables, differences were assessed by chisquared and Fisher's exact tests, as appropriate. A value of *P* < 0.05 was considered to indicate statistical significance.

## 3. Results

There were 21, 18, and 13 patients with type 1 diabetes in Group I, II, and III, respectively, while there were 40 individuals in the control group. All groups had similar gender distribution. Diabetic retinopathy was detected in 59.6% of the diabetic patients. The mean age of patients in group III (diabetics with PDR) was significantly higher than group I (diabetics without retinopathy). The age distribution of the control subjects was similar to the diabetic groups, except for group I, where the mean age was significantly less. The duration of diabetes was significantly longer in group III compared with group I and II (*P* < 0.001). However, no difference was detected between groups I and II (Table 1).

**Figure 1:**
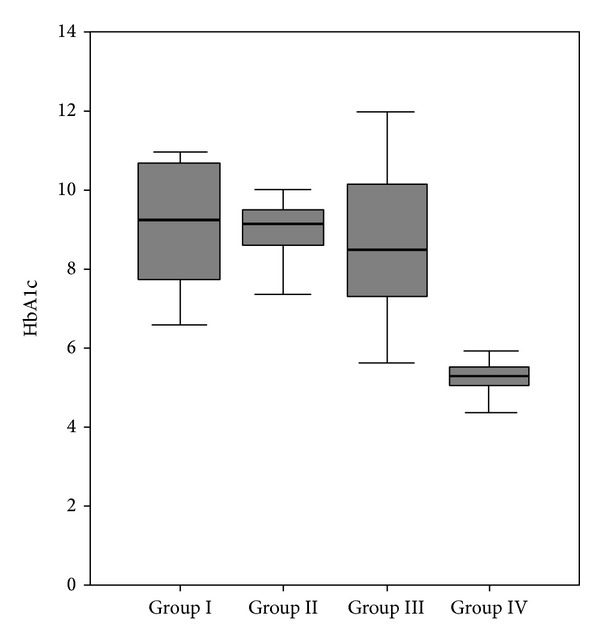
Distribution of HbA1c within the groups.

Microalbuminuria was detected in 12 patients (23.07%) and was predominantly associated with proliferative DR (*n* = 7) but was also observed in patients without retinopathy (*n* = 1) or with nonproliferative retinopathy (*n* = 4). In contrast, macroalbuminuria was observed only in patients with proliferative DR (*n* = 4; 7.67% of the diabetic population). The median serum HbA1c levels were similar in the diabetic groups (group I, II, and III) and were increased significantly, compared with the control group, as expected (*P* < 0.001; Figure 1).

Serum PAI-1 levels were higher in the diabetic groups than control, but this was not statistically significant (*P* = 0.209). The levels of serum fibrinogen (*P* = 0.224) and plasminogen (*P* = 0.244) were similar between the diabetic and control groups (Table 2).

**Figure 2:**
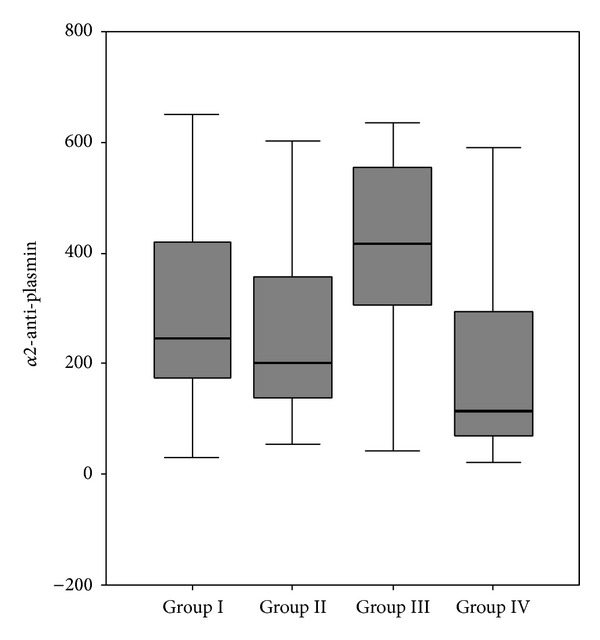
Expression of *α*2-anti-plasmin in the diabetic and control groups.

The levels of alpha-2-anti-plasmin were significantly higher in groups I, II, and III than the control group (*P* < 0.01, *P* < 0.05, and *P* < 0.001, resp.; Figure 2). In addition, a statistically significant positive correlation was identified between serum *α*2-anti-plasmin and HbA1c levels (*r* = 0.268, *P* = 0.031).

## 4. Discussion

In previous studies, it was shown that almost 90% of patients with insulin-dependent diabetes mellitus had retinopathy 10 years after disease onset. After a disease duration of 20 years, this percentage becomes 99%, of which 53% is proliferative and sight threatening [11]. In our study, the prevalence of proliferative retinopathy was positively correlated with disease duration.

The classical factors that are predictive for occurrence of diabetic retinopathy are disease duration, the age at onset and examination, control of diabetes, presence and regulation of hypertension, or proteinuria, and serum creatinine levels. Chronic hyperglycemia during diabetes is the major contributing and well known factor to the occurrence of microvascular complications [12]. Previous studies have demonstrated that the alterations in prothrombotic/inflammatory markers which are related with diabetes and cardiovascular risk develop at an early stage of disease development, and can be partly improved by strict glycemic control [13]. However, the pathogenesis of diabetic retinopathy (DR) is not completely understood, and is suggested to involve inflammation and endothelial dysfunction [14].

The vascular endothelium that constitutes the primary defense against thrombosis is abnormal in diabetes, which might result in the characteristic increased activation of platelets and clotting factors [15]. The pathogenesis of type I DM differs from type II, and there are also differences in the prothrombotic processes. In patients with type I diabetes, the activation of coagulation is the prominent pathway [7, 8], whereas impaired fibrinolysis dominates in type II diabetes [16].

An association between hyperfibrinogenemia and the occurrence of diabetic macroangiopathy was reported [17, 18]. In a study by Asakawa et al., increased fibrinogen levels were reported as a risk factor for retinopathy in type II diabetes patients [19]. Fibrinogen levels were also found to be increased significantly in patients with background retinopathy.

However, the relationship between fibrinogen levels and microangiopathy remains unclear and there are conflicting reports in the literature. For instance, Hirano et al. did not detect differences in fibrinogen levels between diabetic patients with normo-, micro-, and macroalbuminuria [20]. Unlike previous studies, our data purely included patients with type I diabetes, and we did not detect any differences in serum fibrinogen levels between groups.

Elevated serum concentrations of PAI-1 are frequently observed in patients with diabetes, particularly those with type 2 diabetes [21]. Metabolic derangements such as hyperglycemia and hypertriglyceridemia occur due to insulin deficiency and may contribute to the elevated concentrations of PAI-1 observed* in vivo* [22, 23]. However, reports on plasma levels of PAI-1 in type 1 diabetes are contradictory. In the present study, serum PAI-1 levels were slightly higher in the diabetic groups than control, but the differences were not statistically significant (*P* = 0.209). In a previous study of 35 patients, the basal PAI-1 levels of patients with type 1 diabetes were higher than the control group. In contrast, Kırmızıbekmez et al. reported that there was no correlation between serum PAI-1 levels and disease duration or the presence microvascular complications in eighty-four Turkish type 1 diabetic children with a disease duration of more than 5 years [24] which is consistent with our data and a previous study [6].

We observed that serum plasminogen levels were similar between the diabetic subgroups and control. In contrast, levels of *α*2-anti-plasmin were increased significantly in the diabetic groups, compared with control, and were correlated with HbA1c measurement. Although serum *α*-anti-plasmin level was higher in Group III compared to other diabetic subgroups (Group I and II), the difference was not statistically significant. To our knowledge, there are only a small number of studies evaluating *α*2-anti-plasmin levels in Type 1 diabetes, although findings are consistent with our observations. In a recent study, the plasma plasmin-*α*2-anti-plasmin complex (PAP) was associated with diabetic retinopathy and sight threatening diabetic retinopathy in Type 2 diabetics [10], and correlation was still significant after adjustments of the groups for age, gender, race, the study center, SBP, the use of diabetes medications, disease duration, HbA1c, and waist-to-hip ratio were made [25, 26]. A positive correlation between *α*2-anti-plasmin with HbA1c suggests that fibrinolytic markers may improve with disease regulation and better glycemic control.

## 5. Conclusions

Diabetes is a state associated with increased risk for thrombosis and it does not only contribute to major vessel diseases but also to microvascular complications. However, additional studies on larger patient populations are necessary to reveal the fibrinolytic and thrombotic status of patients with Type 1 diabetes. Confirmation of these data would allow a better understanding of the pathogenesis of DR, which may lead to the development of therapies that prevent its onset and/or progression.

## Figures and Tables

**Table 1:** Demographic data of the participants.

Variables	Group I (*n*: 21)	Group II (*n*: 18)	Group III (*n*: 13)	Group IV (*n*: 40)	*P* value
Age (years)	27.3 ± 7.4^a,b^	31.3 ± 9.9	40.2 ± 12.1^a^	35.6 ± 7.7^b^	<**0.001**
Sex					0.391
Female	10 (47.6%)	13 (72.2%)	7 (53.8%)	20 (50.0%)	
Male	11 (52.4%)	5 (27.8%)	6 (46.2%)	20 (50.0%)	
Duration of DM (years)	7 (5–20)^a^	8 (5–32)^c^	21 (5–40)^a,c^	—	**0.002**

^a^Difference between Group I and Group III is significant (*P* < 0.001). ^b^Difference between Group I and Group IV is significant (*P* = 0.006). ^c^Difference between Group II and Group III is statistically significant (*P* < 0.001).

**Table 2:** Biochemical and hemostatic measurements of diabetic patients and healthy controls.

Variables	Group I (*n*: 21)	Group II (*n*: 18)	Group III (*n*: 13)	Group IV (*n*: 40)	*P* value
HbA1c	9.2 (6.6–11.0)^a^	9.2 (6.0–11.0)^b^	8.5 (5.6–12.0)^c^	5.3 (4.4–6.3)^a,b,c^	<**0.001**
PAI	182.7 (9.4–271.0)	158.0 (7.9–255.2)	162.1 (7.9–246.7)	155.6 (107.2–236.9)	0.209
*α*2anti-plasmin	245.0 (31.0–650.0)^a^	202.0 (55.0–904.0)^b^	418.0 (42.0–1184.0)^c^	115.5 (23.0–591.0)^a,b,c^	**0.004**
Plasminogen	90.0 (69.0–152.0)	102.0 (55.0–141.0)	107.0 (57.0–133.0)	87.0 (68.0–121.0)	0.244
Fibrinogen	259.8 ± 72.6	277.3 ± 62.8	307.1 ± 81.3	272.1 ± 42.3	0.224

^a^Difference between Group I and Group IV is significant (*P* < 0.01). ^b^Difference between Group II and Group IV is significant (*P* < 0.05). ^c^Difference between Group II and Group IV is significant (*P* < 0.001).
